# Confronting the uncertainty: Vulnerability to climate change among smallholder farmers in the Sidaama region, Ethiopia

**DOI:** 10.1371/journal.pone.0323469

**Published:** 2025-05-14

**Authors:** Abera Argo Lankamo, Dayanandan R, Bantyergu Engida Bati, Samuel Jilo Dira

**Affiliations:** 1 College of Business and Economics, Hawassa University, Hawassa, Sidaama, Ethiopia; 2 College of Social Science and Humanities, Hawassa University, Hawassa, Sidaama, Ethiopia; University of Agriculture Faisalabad, PAKISTAN

## Abstract

Smallholder farmers’ vulnerability to climate change varies due to socio-economic and biophysical factors, requiring a context-specific assessment. This study examines livelihood vulnerability in the face of climate change in the Sidaama Region, Ethiopia. A mixed-methods approach with a descriptive and explanatory sequential design was employed. Data from 391 systematically sampled households were analyzed using the Livelihood Vulnerability Index (LVI) and the Intergovernmental Panel on Climate Change (IPCC) framework (LVI_IPCC), alongside Kruskal-Wallis and Dunn’s tests. Results of LVI analysis indicate that the Lowland agroecological zone (AEZ) is the most vulnerable (0.466), followed by the Highland (0.412), while the Midland is least vulnerable (0.376). The Highland AEZ showed greater sensitivity to climate change, whereas the Lowland had the weakest adaptive capacity. The Kruskal-Wallis H test confirmed significant differences in vulnerability across AEZs (H = 49.083, *p* < 0.001), with Dunn’s test revealing that the Lowland AEZ is significantly more vulnerable than both the Highland and Midland. LVI_IPCC results similarly ranked the Lowland as the most vulnerable (-0.0041), followed by the Midland (-0.072), with the Highland being the least vulnerable (-0.096). Boxplot analysis further confirmed that the lowland had the highest median LVI_IPCC, indicating greater livelihood vulnerability, while the Highland and Midland had lower median values. To reduce vulnerability, targeted interventions such as climate-smart agriculture, diversified income sources, improved microfinance access, and tailored climate adaptation strategies are needed. Local, regional, and national governments should prioritize disaster prevention and mitigation in the Lowland while leveraging the Midland’s higher adaptability for piloting innovative adaptation strategies.

## 1. Introduction

Smallholder farmers’ vulnerability to climate change varies based on social, economic, and biophysical factors, including agroecological zones and geographical locations [1,2]. Vulnerability is context-specific and shaped by interactions among climate, agricultural practices, and socio-political factors [2]. The Intergovernmental Panel on Climate Change (IPCC) defines vulnerability as “the degree to which a system is susceptible to and unable to cope with adverse effects of climate change, including climate variability and extremes” [3]. Three key dimensions define climate vulnerability: exposure, sensitivity, and adaptive capacity.

Exposure refers to the extent of direct contact with climate stressors [4], while sensitivity reflects how significantly a system is affected, either positively or negatively, by climate fluctuations [5]. A system’s sensitivity to climate change indicates how much it is affected by climate fluctuation or change, whether positively or negatively. A shift in agricultural output in response to variations in the mean, range, or variability of temperature is an example of an immediate impact. Indirect effects include damages resulting from an increase in the frequency of flooding caused by sea level rise [4]. Sensitivity quantifies a system’s response to external stimuli and the potential effects of climate change on it as it exists today. Consequently, a system that is sensitive to climatic changes is highly responsive to it and can be affected by even slight variations in the environment [5]. Adaptive capacity determines how well a system can anticipate, recover from, or mitigate climate-related impacts [4].

Early vulnerability studies followed a “single stressor, single outcome” approach, focusing primarily on physical damages [6,7]. Recent studies emphasize broader socio-economic and political dimensions, including variations based on age, gender, and institutional factors [8].

The Livelihood Vulnerability Index (LVI), developed by [9] assesses smallholder farmers’ vulnerability by considering exposure to natural hazards, adaptation capacities, and sensitivity to climate change impacts [10]. Climate vulnerability assessments (CVAs) help identify vulnerabilities and inform risk reduction strategies [4]. Smallholder farmers are particularly vulnerable due to their economic status, reliance on natural resources, and policy limitations restricting adaptation [11].

Sub-Saharan Africa is among the regions most affected by climate change [12]. Smallholder farmers, practicing predominantly subsistence rain-fed agriculture, have limited means to mitigate and adapt to climate variability [13]. Ethiopia exemplifies these challenges, experiencing severe impacts on its agricultural sector due to reliance on rain-fed farming and limited adaptive capacity [14]. Although Ethiopia has made significant progress in economic development, reducing the rural poverty rate from 30.4% in 2009/10 to 25.6% in 2020 [15], climate-induced vulnerabilities persist. Factors such as conflict, food insecurity, locust invasions, and inflation exacerbate rural poverty [16]. The 2022 drought, the worst in four decades, heightened food insecurity, malnutrition, and poverty [17].

In the Sidaama region, smallholder farmers face multiple climate-related and socio-economic vulnerabilities. The region has a high population density (674 people/km²) [18], with areas like Shebedino District reaching 1,343 people/km². Rural land fragmentation leaves most households with less than one hectare [19], and issues such as soil infertility, acidity [20]; and environmental degradation [21] further threaten livelihoods. Chronic unemployment, droughts, and floods affect districts such as Boricha, Loka Abaya, and Hawassa Zuriya, leading to persistent crop failures, livestock losses, and increased incidences of waterborne diseases [22]. Tropical diseases and pest outbreaks further stress rural livelihoods [23].

National-level studies on smallholder farmers’ vulnerability, such as Zeleke et al. [24], provide broad insights but lack localized specificity. Previous studies focused on limited areas, mainly in northern [25,26], eastern, e.g., Zeleke et al. [24], and central, e.g., Etana et al. [27] parts of Ethiopia. Research in the southern region, including Sidaama, remains scarce. Given that vulnerability varies across regions and contexts [28], a localized assessment is necessary. Existing studies, such as [29] focus on specific drought-prone districts, limiting broader regional insights.

This study explores the livelihood vulnerability of smallholder farmers in Sidaama to climate change and variability, assessing exposure, sensitivity, and adaptive capacity. The main objective is to evaluate household-level vulnerability and analyze differences across agroecological zones. Findings will inform policy recommendations for government agencies, non-governmental organizations, and international development partners to enhance climate resilience in the region.

## 2. Conceptual framework of vulnerability in the study area

The examination of vulnerability to climate variability and extreme events is approached through three key assessments: socio-economic, biophysical, and integrated. Researchers have conceptualized vulnerability in various ways. The socio-economic approach focuses on identifying households’ socio-economic and political status, which is crucial for risk [30]. This approach considers factors such as education, gender, wealth, health, access to credit, technology, infrastructure, social capital, and political power. However, it overlooks environmental factors, which can cause households with similar socio-economic characteristics to experience different levels of vulnerability [30].

The biophysical approach, on the other hand, evaluates the extent of damage environmental stressors inflict on social and biological systems [31]. It is widely applied in natural hazard and climate change studies, assessing the impacts of the biophysical environment on human populations [30].

The integrated vulnerability assessment combines socio-economic and biophysical approaches to provide a more comprehensive understanding of vulnerability [32]. The Intergovernmental Panel on Climate Change [33] supports this integrated perspective, defining vulnerability as a function of exposure, sensitivity, and adaptive capacity. This study adopts that framework, assessing vulnerability based on these three dimensions.

Vulnerability can be interpreted in two ways: outcome vulnerability and contextual vulnerability. Outcome vulnerability is a linear function of projected climate change impacts on a given exposure unit, mitigated by adaptation measures. In contrast, contextual vulnerability takes a multidimensional, process-based view, emphasizing the interactions between climate and society [34]. Given the social-ecological diversity across agroecological zones (AEZs) in the study area, this research follows a contextual approach.

**Fig 1:**
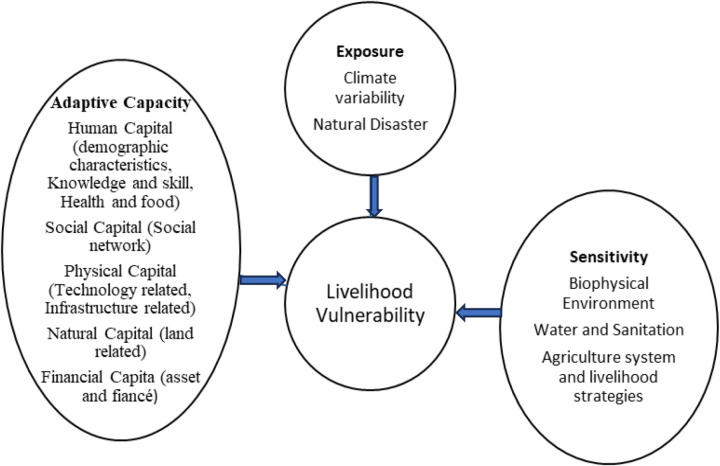
Conceptual framework of livelihood vulnerability.

The unit of analysis for the survey is the smallholder household, defined as “a group of people, each with different abilities and needs, who live together most of the time and contribute to a common economy, sharing food and other income” [35]. Community-level vulnerability, however, is analyzed qualitatively. The conceptual framework guiding this study integrates exposure, sensitivity, and adaptive capacity. Fig 1 illustrates the key components used to develop both the Livelihood Vulnerability Index (LVI) and the IPCC-LVI, highlighting the interactions between these factors.

## 3. Materials and methods

### 3.1 Description of the study area

**Fig 2:**
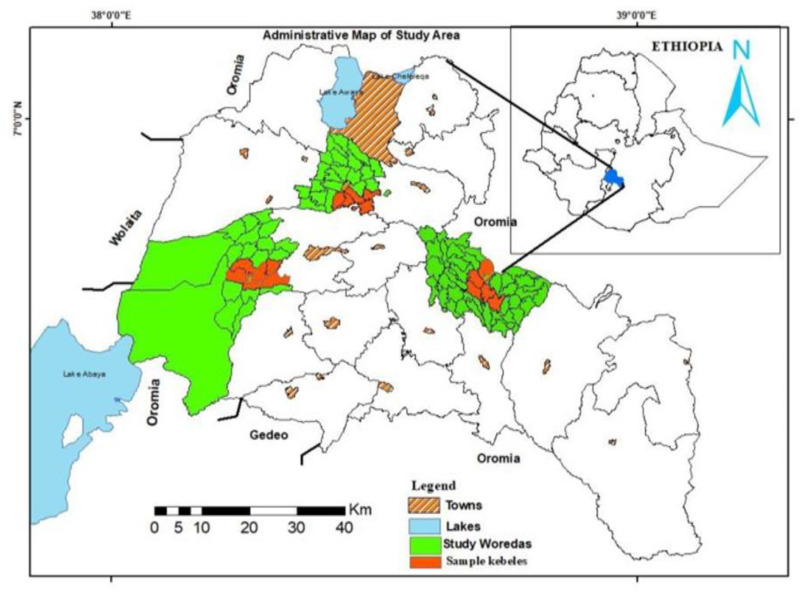
The administrative map of the study area.

This research was conducted in the Sidaama National Regional State (hereafter, Sidaama region), one of Ethiopia’s 12 regional states. Located in the south-central part of the country, it borders the Oromiya and South Ethiopia regions (Fig 2). Geographically, it lies between 6′14″–7′18″ N latitude and 38′20″–39′20″ E longitude, covering 6,539 km² with 30 districts, six town administrations, and Hawassa City Administration. The population is estimated at 5,301,868, growing at 2.9% annually, making it one of Ethiopia’s most densely populated regions, with 633 people per square kilometer [36].

Traditionally, Sidaama is divided into three agroecological zones: Highland *(Alicho*) (15% of the land) at 2,500–3,368 m.a.s.l.; Midland (*Woricho*) (54%) at 1,500–2,500 m.a.s.l.; and Lowland (Gammoojje) (50%), a semi-arid area at 500–1,500 m.a.s.l. [18].

The region’s economy is predominantly subsistence agriculture, with *waasa* as the staple food. Major cash crops include coffee and khat, with Sidaama Coffee renowned for its high-quality Arabica beans. Other crops include barley, wheat, maize, teff, legumes (beans, peas, haricot beans, soybeans), and various fruits (avocado, mango, banana, apple) and vegetables. Sidaama is also known for its strong agricultural cooperatives and coffee production [18].

### 3.2 Research design and approaches

Design decisions typically depend on research strategies, choices, and time horizons [37]. This study employs a cross-sectional design, collecting data from households and community members at a single time. Both descriptive and explanatory sequential research designs were utilized. Grover [38] identifies three research approaches based on philosophical worldview, design, and methods: (1) quantitative (positivism and post-positivism), focusing on measurement and numbers; (2) qualitative (constructivism and transformative), emphasizing words and images; and (3) mixed methods (pragmatism), integrating both. This study adopts a mixed-methods approach, collecting quantitative data first, followed by qualitative data for triangulation.

### 3.3 Sampling and data collection

#### 3.3.1 Sampling.

The study employed a multi-stage sampling procedure, integrating both purposive and systematic sampling methods. To ensure agroecological representation, purposive sampling selected study districts based on variations in vulnerability to climate change and livelihood systems. The selected districts included Arbegoona from the highland (*Alicho*) agroecology, Shebedino from the midland (*Woricho*), and Loka Abaya from the lowland (*Gammoojje*).

Each district reflects distinct livelihood systems: Arbegoona primarily grows *enset*, barley, and wheat; Shebedino focuses on cash crops such as coffee and khat; and Loka Abaya predominantly cultivates maize and haricot beans. These three districts represent 10% of the rural districts in the region, ensuring adequate and relevant information for the study.

To maintain agroecological specificity, the study purposefully selected three kebeles (smallest administrative unit) from each district, ensuring that each belonged exclusively to a single agroecological zone. This approach prevented mixing different agroecological zones within a district. The total sample included households from 11,955 rural households. Using systematic random sampling, the study selected sample households, as household lists were readily available in each kebele.

The sample size was determined using the formula of sampling technique given by [39] as follows.


$$n=\frac{Z^{2}*p*q*N}{e^{2}(N-1)+Z^{2}*p*q}$$

Where,

n=the required sample size,

z = 1.96 (95% confidence interval);

p = the estimated proportion of an attribute that is present in the population, 0.5;

q =1-p;

e= the desired level of precision or error margin=0.05 (5%);

N=total number of smallholder households in selected AEZs= 11955


$$n=\frac{1.96*1.96*0.5*0.5*11955}{0.0025(11955-1)+1.96*1.96*0.5*0.5}\;=\;372.2$$

The sample size determination formula yielded a minimum required sample of 372 households. To account for potential non-responses and incomplete surveys, the final sample size was increased by 5%, resulting in 391 households. The study then allocated sample households across agroecological zones and kebeles using the probability proportional to size (PPS) method to ensure equal representation from each kebele.


$$n_{i}=\frac{n\times n_{i}}{\sum N_{i}}$$

Where n = determined sample size of the research used, $n_{i}$ = HHs of the i^th^ kebele, and $N_{i}$ = total HHs of the i^th^ kebele. Accordingly, a total of 209 (53%) households were systematically selected from midland AEZ, followed by 100 (26%) from highland AEZ, and the remaining 82 (21%) from lowland AEZ.

#### 3.3.2 Data collection.

Standardized and structured interviews were prepared and administered face-to-face using Kobo-Collect via Kobo-Toolbox to collect data from smallholder farm households. Nine enumerators were selected and trained to use Kobo-Collect on smartphones and understand the tool well, emphasizing obtaining household consent before starting the interviews. Data collection occurred from November 9, 2023, to December 16, 2023. Nine focus group discussions (FGDs), three from each district/AEZ, were conducted with community leaders, women and youth groups, agricultural development agents, health extension workers, religious leaders, school principals, and others, selected purposefully in consultation with Kebele administration and development agents (DAs).

Additionally, key informant interviews (KIIs) were conducted with government officials (Kebele, district, and regional levels) and NGO staff operating in the respective districts. FGD and KII guides were used to structure the sessions. Qualitative data was collected from January 1, 2024, to April 30, 2024. The research team also made periodic non-participant observations of the households, communities, and surrounding environments using an observation checklist, supplementing the data from other methods.

### 3.4 Ethical considerations and informed consent

This study involved human subjects through structured interviews, FGDs, KIIs, and non-participant observations. Ethical approval was granted by the Hawassa University College of Business and Economics Research Ethics Committee (approval number: CBE_RTT-11/2019, dated November 2, 2023, protocol version No. 1). Enumerators received training on how to approach respondents, establish rapport, and collect data respectfully and without power imbalances.

Respondents were informed that the study was for academic purposes and that participation was voluntary. They were assured of their right to withdraw at any time and that personal information would remain confidential and anonymous. Informed consent was obtained before the interviews, FGDs, and KIIs, with respondents providing oral consent after understanding the study’s objectives and confidentiality assurances. Consent was documented in Kobo-Toolbox, with the first survey question seeking the respondent’s consent before proceeding. The study did not involve minors, and all personal and demographic data was kept anonymous in the analysis. Results are presented in aggregate form to protect individual identities.

### 3.5 Data analysis methods

The collected data were coded, cleaned in SPSS, and analyzed using descriptive statistics such as percentages, means, and standard deviations to examine the household characteristics of smallholder farmers’ livelihood vulnerability to climate change. Different studies, including [31] and [40] used econometric and indicator-based methods to measure the Livelihood Vulnerability Index (LVI) in the context of disasters and climate change. However, this study employs the LVI developed by [9] which was originally designed to assist planners, policymakers, and development organizations in understanding how physical, social, and demographic factors influence climate vulnerability. [9] emphasized the flexibility of the LVI, allowing it to be tailored to specific regional contexts. Subsequently, modifications were made to fit the local and national contexts of the study area in Sidaama, Ethiopia, in line with the work of [14,30,41]. The LVI used in this study comprises seven major components, further divided into 12 subcomponents to better capture the specific dynamics of the study area. The major components include:

1. **Climate variability and natural disaster (CVND)**, with two subcomponents (climate variability and natural disaster);
2. **Natural environment (NE)**, with three subcomponents (biophysical environment, water and sanitation, agricultural system, and livelihood strategies);
3. **Human capital (HC)**, with three subcomponents (demographic, knowledge and skills, and health and food);
4. **Social capital (SC)**, including social networks, cooperatives, and associations;
5. **Physical capital (PC)**, with two subcomponents (technology-related and infrastructure-related);
6. **Natural capital (NC)** (land-related);
7. **Financial capital (FC)** (assets and access to finance).

The LVI developed for this study is based on the approach outlined by Hahn et al. [9], although several subcomponents and indicators were modified to reflect the specific characteristics of the study region. In total, 66 different indicators were included (see S1 Table). The following methods were used to adjust and standardize these indicators:

1. **Average food diversity index**: The food diversity index, a component of the health and food subcomponent within the human capital major component, was calculated using the Household Dietary Diversity Score (HHDDS) formula developed by [42] and INDDEX [43]. This formula includes 10 food groups, with each group receiving a score of 1 (if consumed in the last 24 hours) or 0 (if not). The average dietary diversity score was then calculated for the study population. For the this study, based on the context of the study area, 10 food groups were selected (cereals; *waasa*/*kocho*; roots and tubers; vegetables; fruits; meat/mutton/poultry/egg; pulses/legumes; milk and milk products; butter/oil/fats; sugar/honey), where the score would range from 0 to 10 and is equal to the total number of food groups consumed by the households The standardization was performed using the inverse of the UNDP’s life expectancy calculation formula since a better dietary diversity score (i.e., higher food diversity) indicates improved adaptive capacity.
2. **Average agricultural landholding**: The average landholding per agroecological zone was calculated and standardized using the UNDP’s life expectancy calculation formula.
3. **Natural disaster variables**: For chronic crop failure, drought, flooding, human disease outbreaks, and livestock disease outbreaks, the questionnaire was based on a three-scale Likert response format. The mean was calculated for each variable, and the standardization was carried out using the UNDP’s life expectancy formula, where responses range from 1 to 3.
4. **Average number of food surplus and adequate months**: The respondents were asked about the number of surplus, adequate, and deficit months of food availability in the past year. Only food surplus and adequate months were considered, as the food deficit months were excluded from the analysis due to their negative implications on adaptive capacity. The average was calculated for the agroecological zones and standardized using the inverse of the UNDP’s life expectancy formula.
5. **Infrastructure access**: Data on access to various infrastructure (e.g., secondary school, domestic water, health center, savings and credit associations, microfinance institutions, and veterinary services) were collected in terms of walking distance. These distances were converted to hours and categorized as “good access” (≤ 0.5 hours), “moderate access” (0.51–1 hour), and “poor access” (>1 hour), in alignment with international standards for infrastructure access (e.g., UNICEF and WHO [44]).
6. **Crop diversification**: This indicator measures the number of crops produced by smallholder farmers. A binary response format was used, where “1” represents crop diversification and “0” represents non-diversification. Eight crop categories were considered, including cereals, weese (false banana), coffee, khat, eucalyptus trees, vegetables, roots, and fruits. The crop diversification index was calculated using the formula developed by [45]. The formula used is indicated in equation (1):

$${div}_{it}={\left(\frac{n_{it}}{N_{jt}}\right)}^{2}$$ (1)

Where, ${div}_{it}$ is crop diversity index; $n_{it}$ is crops a household grows overall in a given year; and $N_{jt}$ is crops are grown in the AEZ overall.

The indicator calculates how many various crops a household grows overall in a given year ($n_{it}$) about how many different crops are grown in the AEZ overall ($N_{jt}$). One benefit of this method over other index generation techniques is that it may be possible to manage the conditions of the AEZ by using the total number of crops now cultivated there as the index’s denominator. Therefore, the agricultural practices common to a household’s own AEZ are used to quantify crop diversity—or lack thereof—rather than the agricultural activities of households in other AEZs. Families in agronomic zones where just a few crops are permitted are not penalized for cultivating a small number of crops. Due to the ratio nature of this index, lower values denote a household that is more agriculturally specialized to the cropping practices in the AEZ, while higher values indicate a household that is more diversified [45]

#### 3.5.1 Calculation of the livelihood vulnerability index (LVI).

This study uses households as the unit of analysis to measure vulnerability, employing the indicator method. The method quantifies vulnerability through a set of indicators, combining them analytically to determine vulnerability levels [9,46]. Vulnerability is assessed based on seven major components, with adaptive capacity represented by five capitals from the sustainable livelihood framework. These components reflect households’ access to resources, both directly and indirectly. Vulnerability dimensions are scored on a scale from 0 to 1, with equal weight assigned to all subcomponents. Indicators are systematically combined to assess vulnerability [8]. The process begins by selecting indicators, assigning weights, and aggregating them into an index, calculated using a formula adapted from the United Nations Development Program’s (UNDP) life expectancy index [47]. Since the subcomponents are measured on different scales, they are standardized using the indexes in Equation 2


$$I_{a}=\;\frac{S_{a}-S_{\min}}{S_{\max}-S_{\min}}$$ (2)

Where, $I_{a}$ is the standardized value of each indicator, $S_{a}$ is the original subcomponent for household a, $S_{\min}$ is the minimum value of the indicator across all households, and $S_{\max}$ is the maximum value of the indicator across all households. For example, for the indicator “temperature has increased over time,” the percentage of respondents who responded “yes” was taken, and the standardization was made based on the actual value where the minimum is zero and the maximum is 100. But when a subcomponent has a negative relationship with vulnerability or when a higher value is good and has a positive contribution in minimizing vulnerability (like formal educational status), the normalized value for each indicator can be computed by equation (3). For example, the indicator “percent of household heads whose education level is primary education and above” is expected to reduce vulnerability, and it is inversely related to vulnerability. Therefore, equation (3) was used for such types of indicators for standardization.


$$I_{a}=\;\frac{S_{\max}-S_{a}}{S_{\max}-S_{\min}}$$ (3)

After each indicator was standardized, the average value of each component was calculated using Equation (4):


$$M_{a}=\frac{\sum_{a=1}^{n}I_{a^{t}}}{n}$$ (4)

Where $M_{a}$ is one of the seven components of household a, $I_{a^{i}}$ indicates the subcomponents indexed by i, which builds each major component, and n is the number of sub-components of each major component. After obtaining values for each of the seven components, the household-level LVI was obtained by combining these components using Equation (5):


$${LVI}_{a}=\frac{\sum_{i=1}^{7}{wm}_{i}M_{a}}{\sum_{i=1}^{7}{wm}_{i}}$$ (5)

Which can be further expressed as:


$${LVI}_{a}=\frac{W_{CVND}{CVND}_{a}+W_{NE}{NE}_{a}+\;W_{HC}{HC}_{a}+\;W_{SC}{SC}_{a}+W_{PC}{PC}_{a}+W_{NC}{NC}_{a}+\;W_{FC}{FC}_{a}}{W_{CVND}+\;W_{NE}+W_{HC}+W_{SC}+W_{PC}+W_{NC}+W_{FC}}$$ (6)

Where ${LVI}_{a}$ is the livelihood vulnerability index for household a, which equals the weighted average of seven major components${,\;wm}_{i}$. The weights of each major component are denoted by the number of subcomponents that make up each major component, which is used to guarantee that all major components have equal contributions to the total LVI. The LVI value ranges between 0 and 1, where 0 denotes the least vulnerable while 1 implies the most vulnerable [8–10].

#### 3.5.2 IPCC framework for measuring livelihood vulnerability.

The IPCC offers an alternative method for calculating the Livelihood Vulnerability Index (LVI) by grouping the seven major components of [9] into exposure, adaptive capacity, and sensitivity. Livelihood vulnerability is defined by the IPCC as a function of system exposure, sensitivity, and adaptive capacity, without specifying their interrelationship [8,48]. In this study, a similar approach is adopted, categorizing the modified components into these three factors. Primary data are used to measure the subcomponents of each factor. The major components of the LVI-IPCC that contribute to vulnerability include exposure (climate variability and natural disasters), adaptive capacity (human capital—demographics, knowledge, skills, health, and food; social capital—social networks, cooperatives, and associations; physical capital—technology and infrastructure; natural capital—land; and financial capital—assets and access to finance); and sensitivity (natural environment—biophysical environment; water and sanitation; and agricultural systems and livelihood strategies). These components are combined in Equation 7.


$${CF}_{b}=\frac{\sum_{i=1}^{n}W_{mi}M_{bi}}{\sum_{i=1}^{n}W_{mi}}$$ (7)

Where:

${CF}_{b}$ is an IPCC-defined contributing factor (exposure, sensitivity, or adaptive capacity) for the agroecology b,${\;M}_{bi}$ is the major component of the agroecology b, indexed by i, $W_{mi}$ is the weightage of each major component, and n is the number of major components in each contributing factor. Once exposure, sensitivity, and adaptive capacity are calculated, the three contributing factors will be combined using Equation (8).


$${LVI\_IPCC}_{b}=(eb-ab)*sb$$ (8)

Where ${LVI-IPCC}_{b}$ is the LVI for i_s_ ${AEZ}_{b}\;$stated in the IPCC vulnerability framework, eb is the computed exposure score for${AEZ}_{b}$, ab is the computed adaptive capacity score for$\;{AEZ}_{b}$, and sb is the calculated sensitivity score for${AEZ}_{b}$. The LVI-IPCC index is scaled from -1 (least vulnerable) to 1 (most vulnerable). It is mostly used as an estimate of the relative vulnerability compared to the populations in agroecology [49,50]. Hence, for the analysis of the LVI for the current study, both LVI and IPCC-LVI are used.

In addition to the LVI and IPCC-LVI indices, inferential statistics were employed following [51] to complement the analysis. Since the LVI is a continuous variable that does not follow a normal distribution, a Kruskal-Wallis H test, a non-parametric alternative to one-way ANOVA, was conducted. Dunn’s pairwise post hoc test with Bonferroni correction was applied to identify significant differences in livelihood vulnerability across agroecological zones. Given the three pairwise comparisons, the Bonferroni correction adjusted the significance threshold to 0.0167 (α = 0.05/3). Thus, only differences with p < 0.0167 were deemed significant. These non-parametric methods are recognized as effective for analyzing non-normal or ordinal data [51,52].

The contributing factors, major and subcomponents, and number of indicators included in the study are illustrated in Table 1.

**Table 1:** IPCC Contributing factors, major and subcomponents, and number of indicators.

Contributing factors	Major components	Subcomponents	Number of indicators
Exposure	Climate variability and natural disaster	Climate variability	7
		Natural disaster	7
Sensitivity	Natural environment	Biophysical environment	3
		Water and sanitation	4
		Agricultural system and livelihood strategies	4
Adaptive capacity	Human capital	Demographic	4
		Knowledge and Skill	4
		Health and food	8
	Social capital	Social networks, cooperatives, and associations	4
	Natural capital	Land-related	3
	Physical capital	Technology-related	5
		Infrastructure-related	7
	Financial capital	Assets and finance	6
***Total***			***66***

## 4. Results and discussions

This section presents the results and discussion of the study, covering the sociodemographic characteristics of respondents and livelihood vulnerability at both household and agroecological zone (AEZ) levels. The analysis was conducted for each contributing factor—**exposure, sensitivity**, and **adaptive capacity**—as well as for the seven major components of vulnerability. The overall LVI and LVI-IPCC scores were also computed and compared across AEZs. To statistically assess differences in livelihood vulnerability among AEZs, the Kruskal-Wallis H test was applied. Moreover, Dunn’s pairwise post hoc test with Bonferroni correction was used to determine which AEZs significantly differed in terms of livelihood vulnerability.

No missing data were recorded, as the Kobo-Toolbox survey design restricted item skipping by making responses mandatory. The following subsections present detailed findings, starting with the sociodemographic characteristics of respondents (Table 2).

**Table 2:** Sociodemographic characteristics of the respondents.

Variables	Description	Frequency	Percent	Mean	Min	Max
Age of the respondents	20-29	12	3.1	43.9 (10.78) ^*^	20	75
	30-39	134	34.3			
	40-49	141	36.1			
	50-59	57	14.6			
	60 and above	47	12.0			
Sex of the respondents	Male	305	78.0			
	Female	86	22.0			
The education level of the HH heads	Uneducated	91	23.3			
	primary (1–6)	153	39.1			
	junior secondary (7–8)	86	22.0			
	secondary (9–12)	33	8.4			
	TeVT (level 1–4)	8	2.0			
	Diploma and above	20	5.1			
Household size	1-3	56	14.3	5.48 (1.89) ^*^	1	13
	4-6	230	58.8			
	7-9	92	23.5			
	10 and above	13	3.3			
Farming activity	Only Crop production	68	17.4			
	Only livestock rearing	6	1.5			
	Mixed farming (crop production and livestock rearing)	317	81.1			
Landholding (ha)	0.25 and below	70	17.9	0.93 (0.60) *	0.001	2
	0.251 to 0.5	94	24.0			
	0.51 to 1.0	108	27.6			
	1.01 to 1.5	54	13.8			
	1.51 and above	65	16.6			

### 4.1 Sociodemographic characteristics of the respondents

This subsection provides an overview of respondents’ demographic characteristics (Table 2).

**Age:** The mean age of household heads is 43.9 years, with 73.5% under 50, indicating that most are in the productive age group (15–64), which is generally associated with lower vulnerability.

**Sex:** Male-headed households are often considered less vulnerable due to greater exposure to formal and informal knowledge. In the study area, 78% of respondents are male, reflecting a predominantly patriarchal society.

**Education:** Education is expected to reduce vulnerability, with household heads who completed primary school or higher facing lower risks. In this study, 39.1% of respondents completed primary education, 22% junior secondary, and 8.4% secondary education, while 23.3% are uneducated, indicating that most respondents have some level of formal education.

**Household Size:** Larger households with more dependents tend to be more vulnerable due to higher food, education, and healthcare costs. The majority (58.8%) have 4–6 members, with an average household size of 5.48, slightly below the national average of 5.55 [53]. Household sizes range from 1 to 13 members, indicating that most households are relatively large.

**Landholding** refers to the total farmland area (in hectares) owned by households. Larger landholdings generally enhance adaptive capacity, as they allow for higher production and lower vulnerability to climate change and variability. The results indicate that 17.9% of households own 0.25 hectares or less, which is minimal and may reduce resilience. Meanwhile, 24% and 27.6% own between 0.251–0.5 and 0.51–1.0 hectares, respectively. Additionally, 13.8% and 16.6% possess 1.01–1.5 and more than 1.51 hectares, respectively. The mean landholding is 0.93 hectares (SD = 0.60), closely aligning with the national average of 0.96 hectares [54]. Overall, about 70% of households own one hectare or less, which may contribute to livelihood vulnerability.

**Farming Activities** encompass crop production and livestock rearing, essential for livelihood diversification. Households practicing mixed farming are expected to be more resilient to climate change impacts. The findings show that the vast majority (81.1%) engage in mixed farming, while 17.4% focus solely on crop production and 1.5% on livestock rearing. This suggests that most respondents have diversified farming practices, potentially improving their livelihood security.

### 4.2 Results of livelihood vulnerability index (LVI) and LVI_IPCC

This section examines smallholder farmers’ livelihood vulnerability to climate change across agroecological zones (AEZs) in the Sidaama region using composite indices. Household-level vulnerability was assessed based on data from three AEZs, incorporating seven major and 12 subcomponents of the LVI (Table 1). Indicator grouping followed the sustainable livelihood framework [55]) and was adapted from Asfaw et al. [14], Simane et al. [49], with modifications for local relevance. Vulnerability was analyzed through three key determinants identified by the IPCC: exposure, sensitivity, and adaptive capacity [56].

#### 4.2.1 Smallholder farmers’ livelihood vulnerability.

**Fig 3:**
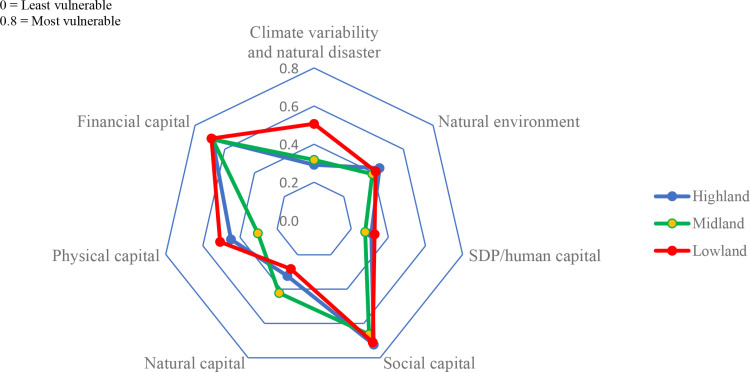
LVI spider diagram of major components. Source: Field survey, 2023.

Table 3 and Fig 3 present the LVI of each major component for each agroecological zone.

**Table 3:** Results of the livelihood vulnerability index.

Main components/ capitals	LVI for each component			Indicators
	Highland	Midland	Lowland	
Climate variability and natural disaster	0.291	0.318	0.507	14
Natural environment	0.44	0.391	0.413	11
Human capital	0.308	0.275	0.326	16
Social capital	0.723	0.664	0.71	4
Natural capital	0.323	0.423	0.283	3
Physical capital	0.448	0.304	0.507	12
Financial capital	0.684	0.682	0.690	6
**LVI**	**0.412**	**0.376**	**0.466**	
**Rank**	**2nd**	**least**	**1st**	

**Exposure: Climate variability and natural disaster:** Lowland (*Gammoojje*) households exhibited the highest vulnerability to climate variability and natural disasters (0.507), followed by Midland (*Woricho*, 0.318) and Highland (*Alicho*, 0.291) (Table 3, Fig 3). This aligns with Berhanu et al. [57], where Lowland areas were found to be more exposed to climate change and variability. In terms of **climate variability**, Lowland farmers faced the highest vulnerability (0.539), followed by Midland (0.324) and highland (0.259). Regarding **natural disasters**, Lowlands remained the most vulnerable (0.473), followed by highlands (0.324) and midlands (0.304) (Table 4).

**Table 4:** Climate variability and natural disaster.

Climate variability						
Indicators	Highland		Midland		Lowland	
	Actual	Index	Actual	Index	Actual	Index
Temperature has increased over time	51	0.51	39.7	0.397	86	0.86
Rainfall has decreased	16	0.16	28.7	0.287	65.9	0.659
Rainfall starts lately	16	0.16	28.2	0.282	53.7	0.537
Early cessation of rainfall	43	0.43	32.1	0.321	30.5	0.305
Spring season (badhessa) rain decreased	22	0.22	26.8	0.268	56.1	0.561
The main rainy season (hawado) rain decreased	5	0.05	26.8	0.268	32.9	0.329
There was erratic rainfall	28	0.28	44.5	0.445	52.4	0.524
**Average index for climate variability**		**0.259**		**0.324**		**0.539**
**Natural disaster**						
**Indicators**	**Highland**		**Midland**		**Lowland**	
Chronic crop failure	1.97	0.485	2.1	0.55	2.49	0.745
Experienced drought	1.8	0.4	1.77	0.385	2.48	0.74
Flooding	1.55	0.275	1.55	0.275	2.04	0.52
Human disease outbreak	1.9	0.45	1.95	0.475	2.01	0.505
Livestock disease outbreak	1.94	0.47	1.88	0.44	2.24	0.62
Injury due to disaster	12	0.12	0	0	15.9	0.159
Death due to disaster	7	0.07	0.5	0.005	2.4	0.024
**Average index for natural disaster**		**0.324**		**0.304**		**0.473**

These findings highlight chronic crop failures, droughts, floods, and disease outbreaks as major natural disaster factors exacerbating the vulnerability of smallholder farmers to climate change and variability.

FGD participants in Arada Gale Kebele, Loka Abaya district, reported experiencing rising temperatures and declining rainfall over the past 5–10 years. They observed erratic rainfall patterns, with delayed onset and early cessation, which adversely affected crop production and livestock rearing. These climatic shifts have increased their exposure to climate change impacts, including drought, flooding, and human and livestock diseases.

**Sensitivity: Natural environment:** The Highland exhibited the highest sensitivity to climate change (0.44), followed by the Lowland (0.431), with the Midland being the least sensitive (0.391) (Table 3, Fig 3). This aligns with [57]], who found the Highland to be most sensitive in their respective studies. However, this contrasts with Zeleke et al. [41]], where the Lowland was most sensitive. The discrepancy may be due to differences in topography and livelihood strategies. The natural environment’s sensitivity was assessed through subcomponents like biophysical environment, water and sanitation, agricultural systems, and livelihood strategies.

**Biophysical environment and water and sanitation:** In terms of the biophysical environment, the Highland showed the highest sensitivity (0.793), followed by the Midland (0.596), with the Lowland showing the least (0.398). Key contributing factors include 87% of Highland households lacking corrugated iron houses, and 76% without early warning systems or access to climate information (Table 5).

**Table 5:** Biophysical environment and water and sanitation subcomponents.

Biophysical Environment			
Indicators	Highland	Midland	Lowland
% of HHs that do not have access to climate-related information	0.75	0.804	0.268
% of HHs that do not get information on early warning	0.76	0.818	0.329
% of HHs not using corrugated iron for the roof of their house	0.87	0.167	0.598
**Average index for the biophysical environment**	**0.793**	**0.596**	**0.398**
**Water and sanitation**			
**Indicators**	**Highland**	**Midland**	**Lowland**
% of HHs not having access to drinking water	0.09	0.139	0.22
% of HHs using water from open/unprotected sources (river, unprotected pond, lake, etc.)	0.23	0.354	0.28
% of HHs not using a pit latrine	0.21	0.062	0.34
% of HHs that use an open pit latrine	0.32	0.005	0.415
**Average index for water and sanitation**	**0.213**	**0.14**	**0.314**

For water and sanitation, sensitivity was lower across all AEZs, but the Lowland exhibited the greatest sensitivity (0.314), followed by the Highland (0.213), and the Midland was the least (0.14). Compared to the other two, households that use open pit latrines are higher in the Lowland, contributed significantly to the sensitivity in this area (0.34). The households that use open pit latrines are almost none in the midland (0.005).

The FGD and KII participants in the Midland (Shebedino district) reported that the district had been free from open defecation for over 10 years, thanks to health extension services and training provided by various governmental and non-governmental organizations. This initiative, recognized by both the district and the region, has contributed to the lower sensitivity of the Midland AEZ to climate change and variability

**Agricultural system and livelihood strategies:** The Lowland exhibited greater sensitivity (0.53) to agricultural systems and livelihood strategies, followed by the Midland (0.437), with the Highland being the least sensitive (0.311) (Table 3 and Fig 3). In the Lowland AEZ, 91.4% of households do not work outside the community, 41.5% do not engage in off-farm activities, and 31.7% do not practice mixed farming. These factors may contribute to the heightened vulnerability to climate change in this area (Table 6). FGD participants in the Lowland attributed this to a lack of awareness and training on off-farm activities, which could provide additional income. Many households also rely on single agricultural activities like crop production or livestock rearing, making them more vulnerable to climate impacts.

**Table 6:** Agricultural system and livelihood strategies.

Indicators	Highland	Midland	Lowland
% of households that do not engage in off-farm activities	0.24	0.36	0.45
% of HHs with members not working outside the community	0.63	0.675	0.915
Average crop diversification index	0.364	0.486	0.420
% of HH that does not engage in mixed farming	0.01	0.225	0.317
**Average index for agricultural system and livelihood strategies**	**0.311**	**0.437**	**0.53**

**Adaptive capacity: Human capital, social capital, physical capital, natural capital, and financial capital:** Adaptive capacity, which reflects smallholder farmers’ ability to adjust to climate change, is measured through five types of capital: human, social, physical, natural, and financial. The subcomponents under adaptive capacity have a negative relationship with vulnerability, or a higher value is good and has a positive contribution to minimizing vulnerability. In this regard, the normalized value for each indicator has been computed by Equation 2 above. Hence, the lower composite index indicates better adaptive capacity, and the higher indicates less adaptive capacity.

**Human capital:** For human capital, the Midland exhibited the best adaptive capacity (0.275), followed by the Highland (0.308), and the Lowland (0.326) (Table 3 and Fig 3). In the Midland, a smaller number of households had disabled members or orphaned children, contributing to its better adaptive capacity. The Midland also scored higher for knowledge and skills (0.337), benefiting from better access to educational institutions and agricultural extension services (Table 7). FGD participants in the Midland highlighted the accessibility of educational programs, which is attributed to the district’s proximity to key roads and Hawassa, the regional capital.

**Table 7:** Sociodemographic characteristics and knowledge and skill.

Sociodemographic characteristics			
Indicators	Highland	Midland	Lowland
% of productive age (age between 15–64 size)	0.415	0.347	0.40
% of male-headed household	0.18	0.278	0.122
% of HHs with no orphaned children	0.24	0.057	0.171
% of HHs with no persons of disability	0.03	0.048	0.049
**The average index of demographic**	**0.216**	**0.182**	**0.185**
**Knowledge and Skill**			
**Indicators**	**Highland**	**Midland**	**Lowland**
% of HH heads whose education level is primary education and above	0.33	0.23	0.354
% of HH heads that have access to agricultural extension service	0.17	0.057	0.012
% HH heads that have a mobile phone	0.51	0.507	0.659
% of HH heads that have access to training	0.65	0.555	0.646
**Average index of knowledge and skill**	**0.415**	**0.337**	**0.418**

For health and food, the Highland exhibited better adaptive capacity (0.325), followed by the Midland (0.343), and the Lowland (0.406). The Highland’s higher use of irrigation (40%) compared to the Midland (27.3%) and Lowland (26.8%) contributed to its better health and food security (Table 8). FGD participants in the Highland mentioned that the cultivation of *weese* (false banana), which is resilient to erratic rainfall, helped mitigate food shortages despite changing weather conditions.

**Table 8:** Health and food subcomponent.

Indicators	Highland	Midland	Lowland
average food diversity index	0.632	0.594	0.618
the average number of food surplus and adequate months	0.392	0.427	0.4367
% of HHs that save crops for difficult times	0.42	0.34	0.488
% of HHs using any irrigation sources	0.6	0.727	0.732
% of HH members not suffering from chronic illness	0.08	0.1	0.146
% of HHs where a family member had not miss school/work in the last one month due to illness	0.08	0.11	0.183
% of HH members that do not suffer from malaria, TB, cholera	0.07	0.086	0.195
% of HHs getting health extension service	0.06	0.057	0.195
**Average index for health and food**	0.291	**0.305**	**0.374**

**Social capital:** The Midland demonstrated the best adaptive capacity in social capital (0.664), followed by the Lowland (0.71), with the Highland exhibiting the least (0.723) (Tables 3 and 9 and Fig 3). Membership in cooperatives, such as those under the Sidaama Coffee Producers’ Union, played a significant role in improving adaptive capacity in the Midland. FGD participants in Shebedino district (Midland) noted that cooperative membership helped strengthen the AEZ’s social capital.

**Table 9:** Social network.

Social networks, cooperatives, and associations			
Indicators	Highland	Midland	Lowland
% of HHs have not visited local government for support within the past 12 months	0.740	0.794	0.805
% of HHs with members having cooperative membership	0.710	0.483	0.573
%HH members that have responsibility in the community	0.700	0.593	0.659
% of HHs with women’s group membership	0.740	0.79	0.805
**Average index for social network**	**0.723**	**0.664**	**0.71**

**Natural capital:** The Lowland exhibited the best adaptive capacity under natural capital (0.283), followed by the Highland (0.323), and the Midland (0.423) (Table 3 and Fig 3). Land possession and the use of soil and water conservation schemes were major contributors to the Lowland’s adaptive capacity (Table 10).

**Table 10:** Natural capital (land-related subcomponent).

Natural Capital			
Indicators	Highland	Midland	Lowland
% of HHs that possess land	0.01	0	0
Average agricultural landholding (ha)	0.364	0..65	0.618
% of HHs using Soil and water conservation Scheme	0.59	0.617	0.232
**Average index for land-related subcomponent**	**0.323**	**0.423**	**0.283**

**Physical capital:** The Midland demonstrated better adaptive capacity (0.304) for physical capital, followed by the Highland (0.448), with the Lowland exhibiting the least (0.507) (Table 3 and Fig 3). Physical capital was assessed based on two subcomponents: technology and infrastructure. For the technology subcomponent, Midland demonstrated the highest adaptive capacity (0.269), surpassing Lowland (0.438), while Highland had the lowest (0.488). Key factors that contributed to Midland’s higher capacity included the use of improved seeds, inorganic fertilizers, and the presence of houses with corrugated iron (Table 11).

**Table 11:** Technology and infrastructure-related subcomponents.

Technology related			
Indicators	Highland	Midland	Lowland
% of HHs using inorganic fertilizer	0.20	0.038	0.354
% of HHs using improved seeds	0.40	0.029	0.39
% of HHs using pest and insecticides	0.370	0.383	0.354
% of HHs using any irrigation sources	0.600	0.727	0.732
% of HHs that have corrugated iron roofed house	0.870	0.167	0.598
**Average index for technology-related**	0.488	**0.269**	0.438
**Infrastructure related**			
**Indicators**	**Highland**	**Midland**	**Lowland**
Access to nearest secondary school (minute)	0.55	0.512	0.939
Access to collect domestic water (minute)	0.21	0.153	0.244
Access to the nearest health center (minute)	0.33	0.268	0.085
Access to nearest saving and credit association (minute)	0.43	0.273	0.646
Access to nearest MFI (minute)	0.38	0.474	0.732
Access to nearest veterinary service (minute)	0.4	0.124	0.305
Access to the nearest main market (minute)	0.56	0.574	0.744
**Average index for infrastructure-related**	**0.409**	**0.340**	**0.528**

In terms of the infrastructure subcomponent, Midland again showed better adaptive capacity (0.340), followed by Highland (0.409), while Lowland had the least (0.528). Important infrastructural factors for Midland included easy access to services such as veterinary care, water, health centers, and savings and credit associations, all of which enhanced its adaptive capacity (Table 11). These findings align with other studies suggesting that Midland has superior adaptive capacity in infrastructure and services [57].

FGD participants from Midland (Shebedino district) highlighted that the quality of housing, particularly the prevalence of corrugated iron houses, varied based on household financial and asset base. However, a significant portion of households in the AEZ had corrugated iron houses, contributing positively to adaptive capacity. During field visits, researchers observed that Midland had more corrugated iron houses compared to Lowland and Highland, which made rural areas in this AEZ appear more developed by Ethiopian standards. Additionally, access to vital services such as veterinary care, healthcare, and credit facilities is notably better in Midland, further enhancing adaptive capacity.

**Financial capital:** Both the Highland and Midland exhibited better adaptive capacity under financial capital (0.68), while the Lowland had slightly lower capacity (0.69) (Table 3 and Fig 3). In these areas, factors such as average crop income, asset value, and access to credit contributed to the adaptive capacity (Table 12). FGD participants in the Midland (Telamo Kantise Kebele) highlighted income from coffee, *khat*, and fruits like avocado and mango as key sources of financial resilience. However, they also mentioned that poor money management hindered further resilience building.

**Table 12:** Asset and financial access.

Indicators	Highland	Midland	Lowland
average value of assets (in Birr)	0.800	0.779	0.789
Average crop income (in Birr)	0.896	0.82	0.869
Average TLU	0.728	0.881	0.778
% of HHs who do not have loan Burdon	0.23	0.033	0.037
% of HHs that save money in financial institutions	0.6	0.636	0.695
% of HHs that have access to credit	0.85	0.943	0.976
**Average index for asset and financial access**	**0.68**	**0.68**	**0.69**

Overall, the results indicated that the Lowland was the most vulnerable AEZ (0.466), followed by Highland (0.412), with Midland showing the least vulnerability (0.375) (Table 3). Similar studies in Ethiopia have also found the Lowlands to be the most vulnerable compared to the Midlands and Highlands [14,41,57].

The Kruskal-Wallis H **test** showed a statistically significant difference in livelihood vulnerability across the three AEZs (H = 49.083, p < 0.001), indicating that at least one AEZ has significantly different vulnerability levels. Dunn’s pairwise comparisons revealed that the Lowland had significantly higher vulnerability than both the Highland and Midland. Specifically, the differences between Lowland and Highland (p = 0.002) and Lowland and Midland (p < 0.001) were statistically significant. However, the difference between Highland and Midland was not significant (p = 0.120) (Table 13), suggesting similar vulnerability levels in these two AEZs.

**Table 13:** Results of Dunn’s pairwise comparisons.

Comparison	Original P-value	sig. at (0.0167) level ^*^	Interpretation
Highland Vs. Lowland	0.002	Significant	Loka Abaya has higher LVI
Highland Vs. Midland	0.120	Not significant	No difference
Midland Vs. Lowland	0.000	Significant	Loka Abaya has higher LVI

*Bonferroni adjusted significance level

#### 4.2.2 LVI_IPCC-based analysis of livelihood vulnerability.

**Fig 4:**
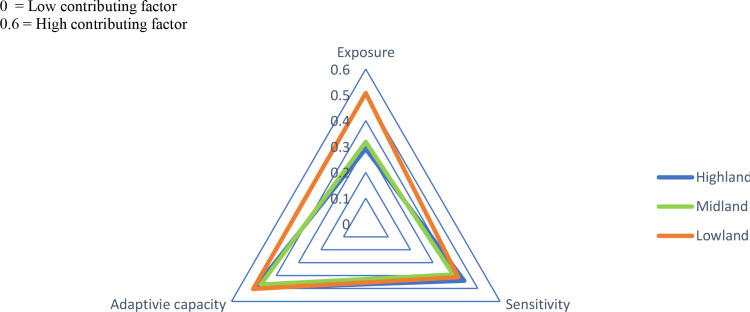
Triangle diagram of contributing factors of LVI_IPCC for the three agroecological zones. Source: Field survey, 2023.

This subsection presents the IPCC-based analysis of livelihood vulnerability, considering the three factors contributing to vulnerability: exposure, sensitivity, and adaptive capacity. The LVI_IPCC measures vulnerability at the AEZ level, where lower exposure, sensitivity, or higher adaptive capacity indicate reduced vulnerability. Table 14 and Fig 4 show the overall LVI_IPCC for each AEZ, with detailed scores for exposure, sensitivity, and adaptive capacity. The triangle diagram ranges from 0 to 0.6, where 0 indicates low and 0.6 indicates high contributing factors. The analysis revealed varying patterns across AEZs (Highland, Midland, and Lowland).

**Table 14:** Results of the LVI_IPCC approach.

Contributing factor	Major components/ capitals	Indicator (NO)	Major component values			Value of contributing factors		
			Highland	midland	lowland	Highland	Midland	Lowland
Exposure	Climate variability and Natural disaster	14	0.291	0.318	0.507	0.291	0.318	0.507
Sensitivity	Natural Environment	11	0.44	0.391	0.413	0.44	0.391	0.413
Adaptive capacity	Sociodemographic/ Human capital	16	0.308	0.275	0.326	0.497	0.469	0.503
	Social capital	4	0.723	0.664	0.71			
	Natural Capital	3	0.323	0.423	0.283			
	Physical Capital	12	0.448	0.304	0.507			
	Financial capital	6	0.684	0.682	0.69			

**Exposure:** The results showed that the Lowland (0.507) is the most exposed AEZ to climate change and variability, followed by Midland (0.318), while the Highland (0.291) is the least exposed (Table 14 and Fig 4). This suggests that the Lowland requires more emphasis on disaster prevention, mitigation, preparedness, response, and recovery. This finding aligns with [14] but contrasts with Berhanu et al. [57], where the Highlands were the most exposed.

LVI_IPCCb = (eb - ab) * sb.

Hence, for Highland LVI_IPCC= (0.291-0.497) *0.44 which is -0.096

**Sensitivity:** The Highland (0.44) was found to be the most sensitive AEZ to climate change and variability, followed by Lowland (0.413) and Midland (0.391) (Table 14 and Fig 4). This indicates greater vulnerability in the Highland, consistent with Berhanu et al. [57].

**Adaptive capacity:** The Lowland exhibited the lowest adaptive capacity (0.503), followed by Highland (0.497), with Midland (0.469) showing the highest adaptive capacity (Table 14 and Fig 4). This is in line with [46], where the Lowland developed less adaptive capacity.

**Overall findings:** The Lowland was the most vulnerable AEZ (-0.0041), followed by Midland (-0.072), with the Highland (-0.096) being the least vulnerable. Both LVI and LVI_IPCC analyses show that the Lowland is more vulnerable, though the second most vulnerable AEZ differs: the Highland is more vulnerable in the LVI analysis, while the Midland is more vulnerable in the LVI_IPCC analysis. This suggests that at the household level, the Highland is more vulnerable, whereas at the AEZ level, the Midland is more vulnerable.

**Fig 5:**
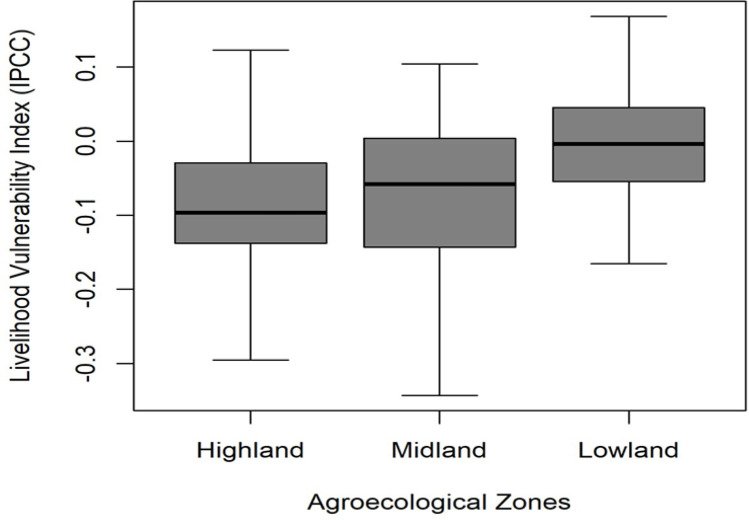
Boxplot of LVI_IPCC across agroecological zones (AEZs).

The boxplot of LVI_IPCC across AEZs also shows that the Lowland has the highest median LVI_IPCC, indicating greater livelihood vulnerability compared to the Highland and Midland. The interquartile range (IQR) is similar across AEZs, indicating comparable variability in vulnerability within each AEZ. However, the Lowland has a slightly higher upper quartile, suggesting that some households experience greater vulnerability than those in the other AEZs (Fig 5).

## 5. Conclusion

A comprehensive assessment of livelihood vulnerability to climate change among smallholder farmers in the Sidaama region was conducted using both the Livelihood Vulnerability Index (LVI) and LVI_IPCC analysis methods. The study highlighted significant vulnerability across the three agroecological zones (AEZs). The Lowland AEZ emerged as the most vulnerable, primarily due to its limited adaptive capacity and high exposure to natural disasters. The Highland AEZ, though exhibiting high sensitivity to climatic variability, was found to be the least vulnerable overall. The Midland AEZ displayed the best adaptive capacity with least vulnerable in terms of LVI measure but faced challenges in exposure.

Results from the Kruskal-Wallis H test indicated statistically significant differences in vulnerability across AEZs (H = 49.083, p < 0.001), with Dunn’s pairwise comparisons confirming that vulnerability in the Lowland was significantly higher than in both the Highland (p = 0.002) and Midland (p < 0.001) AEZs. The Boxplot analysis further revealed that the Lowland had the highest median LVI_IPCC, indicating greater livelihood vulnerability compared to the other AEZs, while the Highland and Midland had lower median values and similar variability in vulnerability.

To mitigate livelihood vulnerability and enhance resilience, tailored interventions such as climate-smart agriculture, income diversification, early warning systems, improved access to microfinance and credit institutions, and enhanced nutritional and health support are essential. Additionally, increased access to food security and higher-quality education are crucial. Each AEZ should develop a customized climate adaptation plan, supported by mechanisms for monitoring and evaluating program success. Community involvement will strengthen resilience and sustainability, helping farmers secure their livelihoods and adapt to challenging climate conditions.

## Supporting information

S1 Table Contributing factors, major and subcomponents, indicators, and hypothesis. (DOCX)
