# Supplementary material for: Confronting the uncertainty: Vulnerability to climate change among smallholder farmers in the Sidaama region, Ethiopia
Source: PLoS One. 2025 May 14;20(5):e0323469. doi: 10.1371/journal.pone.0323469 (PMC12077685; doi:10.1371/journal.pone.0323469)
Supplement: S1 Table — (DOCX) [file pone.0323469.s001.docx]

**S1 Table. Contributing factors, major and subcomponents, indicators, and hypothesis**

| **IPCC factors** | **Major component/capitals** | **Subcomponent** | **Indicators** | **Hypothesis** |
| --- | --- | --- | --- | --- |
| Exposure | Climate variability and natural disaster | Climate variability | Temperature has increased over time | Temperature and rainfall variability are positively related to vulnerability to climate change. |
|  |  |  | Rainfall has decreased |  |
|  |  |  | Rainfall starts lately |  |
|  |  |  | Early cessation of rainfall |  |
|  |  |  | Spring season (badhessa) rain decreased. |  |
|  |  |  | The main rainy season (hawado) rain decreased. |  |
|  |  |  | There was erratic rainfall. | HHs experiencing such disaster conditions are regarded as vulnerable to climate change and the situations are positively related to vulnerability. |
|  |  | Natural disaster | Chronic crop failure |  |
|  |  |  | Experienced drought |  |
|  |  |  | flooding |  |
|  |  |  | Human disease outbreak |  |
|  |  |  | Livestock disease outbreak |  |
|  |  |  | Injury due to disaster |  |
|  |  |  | Death due to disaster |  |
| Sensitivity | Natural Environment | Biophysical environment and information | % of HHs that do not have access to climate-related information | Those HHs with a lack of climate and early warning information and do not have corrugated iron-roofed houses are vulnerable and sensitive to climate change |
|  |  |  | % of HHs that do not get information on early warning |  |
|  |  |  | % of HHs not using corrugated iron for the roof of their house |  |
|  |  | Water and sanitation | % of HHs not having access to drinking water | Those HHs do not have access to drinking water, use an open or unprotected source, do not use pit latrines, and use open pit latrines are vulnerable to climate change. |
|  |  |  | % of HHs using water from open/unprotected sources (river, unprotected pond, lake, etc.) |  |
|  |  |  | % of HHs not using a pit latrine |  |
|  |  |  | % of HHs that use an open pit latrine |  |
|  |  | Agricultural system and livelihood strategies | % of households that do not engage in off-farm activities | HHs that do not engage in off-farm, members do not work outside, specialize in a single crop, and do not engage in mixed farming are sensitive and vulnerable to climate change |
|  |  |  | % of HHs with members not working outside the community |  |
|  |  |  | Average Crop Diversification Index |  |
|  |  |  | % of HH that does not engage in mixed farming |  |
| Adaptive capacity | Sociodemographic/human | Demographic | % of the household heads who are at a productive age level  % of dependents HH members (age below 15 + age 65 and above out of the total HH size) | HH members whose head is at a productive age level, with a low percent of dependents, male-headed, HHs with a smaller number of orphaned children and disabled persons are expected to be better adaptive to climate change |
|  |  |  | % of male-headed household |  |
|  |  |  | % of HHs with no orphaned children |  |
|  |  |  | % of HHs with no persons of disability |  |
|  |  | Knowledge and skill | % of HH members whose education level is primary education and above | HH heads with an education level of primary and above, mobile phone owned, that have access to training and extension service are expected to have better adaptive capacity to climate change |
|  |  |  | % of HH heads that have access to agricultural extension service |  |
|  |  |  | % HH heads that have a mobile phone |  |
|  |  |  | % of HH heads that have access to training |  |
|  |  | Health and food | average food diversity index | HHs with high HHDDS, more months of surplus/adequate food, saving crops for difficult times, using irrigation, not suffering from long-term illness, and getting better health extension services are expected to have better adaptive capacity. |
|  |  |  | the average number of food surplus and adequate months |  |
|  |  |  | % of HHs that save crops for difficult times |  |
|  |  |  | % of HHs using any irrigation sources |  |
|  |  |  | % of HH members not suffering from chronic illness |  |
|  |  |  | % of HHs where a family member had not miss school/work in the last one month due to illness |  |
|  |  |  | % of HH members that do not suffer from malaria, TB, cholera |  |
|  |  |  | % of HHs getting health extension service |  |
|  | Social capital | Social networks, cooperatives, and associations | % of HHs have not visited local government for support within the past 12 months | When the ratio of receiving and borrowing is lower to giving and lending, respectively, does not seek government support, HHs with cooperatives and women's group membership are expected to have better adaptive capacity. |
|  |  |  | % of HHs with members having cooperative membership |  |
|  |  |  | %HH members that have responsibility in the community |  |
|  |  |  | % of HHs with women's group membership |  |
|  | Natural capital | Land related | % of HHs that possess land | HHs that process better land resources and use soil and water conservation (SWC) schemes are expected to have better adaptive capacity. |
|  |  |  | Average agricultural landholding (ha) |  |
|  |  |  | % of HHs using Soil and water conservation Scheme |  |
|  | Physical capital | Technology related | % of HHs using inorganic fertilizer | HHs that use such technologies are expected to have better adaptive capacity. |
|  |  |  | % of HHs using improved seeds |  |
|  |  |  | % of HHs using pest and insecticides |  |
|  |  |  | % of HHs using any irrigation sources |  |
|  |  |  | % of HHs that have corrugated iron roofed house |  |
|  |  | Infrastructure related | Access to nearest secondary school (minute) | HHs nearer to such infrastructures are expected to have better adaptive capacity. |
|  |  |  | Access to collect domestic water (minute) |  |
|  |  |  | Access to the nearest health center (minute) |  |
|  |  |  | Access to nearest saving and credit association (minute) |  |
|  |  |  | Access to nearest MFI (minute) |  |
|  |  |  | Access to nearest veterinary service (minute) |  |
|  |  |  | Access to the nearest main market (minute) |  |
|  | Financial capital | Asset and finance-related | average value of assets (productive and household) | HHs that process better assets and have access to finance are expected to have better adaptive capacity |
|  |  |  | Average crop income |  |
|  |  |  | Average TLU |  |
|  |  |  | % of HHs who do not have a loan burden |  |
|  |  |  | % of HHs that save money in financial institutions |  |
|  |  |  | % of HHs that have access to credit |  |
